# Genomic imbalances in esophageal squamous cell carcinoma identified by molecular cytogenetic techniques

**DOI:** 10.1590/S1415-47572010005000028

**Published:** 2010-06-01

**Authors:** Marilanda Ferreira Bellini, Ana Elizabete Silva, Marileila Varella-Garcia

**Affiliations:** 1 Laboratório de Citogenética e Biologia Molecular, Departamento de Biologia, Universidade Estadual Paulista Júlio de Mesquita Filho', Campus São José do Rio Preto, SPBrazil; 2 Medicine/Medical Oncology, Health Sciences Center, University of Colorado, Aurora, COUSA

**Keywords:** CGH, esophageal carcinoma, FISH, genomic imbalances, molecular cytogenetics

## Abstract

This review summarizes the chromosomal changes detected by molecular cytogenetic approaches in esophageal squamous cell carcinoma (ESCC), the ninth most common malignancy in the world. Whole genome analyses of ESCC cell lines and tumors indicated that the most frequent genomic gains occurred at 1, 2q, 3q, 5p, 6p, 7, 8q, 9q, 11q, 12p, 14q, 15q, 16, 17, 18p, 19q, 20q, 22q and X, with focal amplifications at 1q32, 2p16-22, 3q25-28, 5p13-15.3, 7p12-22, 7q21-22, 8q23-24.2, 9q34, 10q21, 11p11.2, 11q13, 13q32, 14q13-14, 14q21, 14q31-32, 15q22-26, 17p11.2, 18p11.2-11.3 and 20p11.2. Recurrent losses involved 3p, 4, 5q, 6q, 7q, 8p, 9, 10p, 12p, 13, 14p, 15p, 18, 19p, 20, 22, Xp and Y. Gains at 5p and 7q, and deletions at 4p, 9p, and 11q were significant prognostic factors for patients with ESCC. Gains at 6p and 20p, and losses at 10p and 10q were the most significant imbalances, both in primary carcinoma and in metastases, which suggested that these regions may harbor oncogenes and tumor suppressor genes. Gains at 12p and losses at 3p may be associated with poor relapse-free survival. The clinical applicability of these changes as markers for the diagnosis and prognosis of ESCC, or as molecular targets for personalized therapy should be evaluated.

## Esophageal Carcinoma

Cancer of the esophagus is the ninth most common malignancy in the world, but its incidence varies considerably among geographical regions (Lam*,* 2000), with a high incidence in China, Japan, Singapore and Puerto Rico (INCA, 2009a). The American Cancer Society estimated that around 16,470 new cases of esophageal carcinoma occurred in the USA in 2008 (American Cancer Society, 2009). In Brazil, esophageal cancer ranked sixth in cancer mortality in 2000, with 5,307 deaths; about 10,550 new cases were reported in 2008, with an incidence of 1.04-19.07 per 1000,000 males and 0.39-7.58 per 100,000 females (INCA, 2009b).

The development of human esophageal cancer is progressive, involving the accumulation of genetic changes that culminate in malignant transformation (Knudson, 1985; Somers and Schechter, 1992; Xue *et al.*, 2006). An early indicator of this process is the increased proliferation of esophageal epithelial cells that morphologically progresses to basal cell hyperplasia, dysplasia, carcinoma *in situ* and invasive carcinoma (Muñoz, 1997; Mandard *et al.*, 2000; D'Amico, 2006).

With the exception of the USA, esophageal squamous cell carcinoma (ESCC) is histologically the most prevalent type of esophageal cancer worldwide and has a multifactorial origin. In addition to environmental components (Crawford, 2004), several genetic factors are associated with esophageal carcinogenesis, such as chromosomal aneuploidy, allelic deletions, activation of oncogenes and inactivation of tumor suppressor genes (Kuwano *et al.*, 2005). At the cellular level, these factors lead to disorders of cell proliferation, differentiation and apoptosis (Koch *et al.*, 1994; McCabe and Dlamini, 2005; D'Amico, 2006; Daigo and Nakamura, 2008; Khushalani, 2008).

Specific chromosomal aberrations have been identified as markers for the diagnosis and prognosis of solid tumors (Tada *et al.*, 2000; Yen *et al.*, 2001; Shiomi *et al.*, 2003; Qin *et al.*, 2004, 2005a,b, 2008; Wang *et al.*, 2006). Despite reports of numerous chromosomal alterations, no particular diagnostic or prognostic chromosomal markers have been described for esophageal carcinoma. The aim of this review is to summarize the recurrent chromosomal changes, such as gains and losses in regions that may harbor oncogenes and tumor suppressor genes that have been detected in ESCC by molecular cytogenetic approaches. These chromosomal imbalances may represent clinically relevant markers for the diagnosis and prognosis of ESCC or for the identification of novel therapeutic targets.

## Molecular Cytogenetic Technologies Identify Genomic Changes In Cancer

The primary cytogenetic technique used to investigate the molecular pathogenesis of esophageal carcinogenesis is DNA fluorescence *in situ* hybridization (FISH). This technique uses small fragments of DNA as fluorescent probes that bind to specific chromosomal sequences of the target DNA to which they show a high degree of complementarity (Bauman *et al.*, 1980; Langer *et al.*, 1981). FISH probes are often derived from DNA fragments that vary from a few hundred to 200,000 base-pairs; these fragments are isolated, purified, amplified and labeled with fluorochrome-conjugated nucleotides. The DNA probes hybridize to distinct DNA targets such as metaphase chromosomes, interphase nuclei, and extended chromatin fibers and DNA fragments in a variety of biological specimens or platforms, including isolated cells, tissue sections and bacterial artificial chromosomes (BAC) and oligonucleotide arrays (Solinas-Toldo *et al.*, 1997; Pinkel *et al.*, 1998; Speicher and Carter, 2005).

There are numerous variants of the FISH assay, with the most effective for detecting extensive genomic imbalances being comparative genomic hybridization (CGH). CGH was originally developed as the competitive hybridization of a mixture of test DNA and normal reference DNA, labeled with different fluorochromes, to metaphase spreads of a normal specimen, *i.e.*, metaphase CGH (mCGH) (Kallioniemi *et al.*, 1992; du Manoir *et al.*, 1993). In these conditions, chromosomal regions of the test DNA that have normal copy numbers will show a balanced ratio of hybridization with the test and control DNA, whereas chromosomal regions with an excess or loss of copy numbers in the test DNA will show predominantly the color of the hybridization in the normal template. The test DNA used in the CGH assay can be extracted from dividing or non-dividing cells from virtually all types of tissues, including formalin-fixed specimens; the latter possibility allows an informative overview of archived specimens. The level of resolution of specific imbalances in the mCGH assay depends essentially on the condensation of the chromosomes to which the DNA mix is hybridized and ranges from 5 to 10 Mb (Kallioniemi *et al.*, 1992; Speicher and Carter, 2005). Although it is possible to identify large regions involved in low level of genomic gain or loss and small regions with focal amplification, the resolution of the mCGH is generally limited or insufficient for the identification of specific chromosomal bands (Kallioniemi *et al.*, 1992; Speicher and Carter, 2005). Consequently, mCGH is generally used as a preliminary tool to infer potential genes located in imbalanced regions and to confirm or support the results of studies with higher resolution techniques, such as array CGH (aCGH) or FISH with single gene probes (Nakakuki *et al.*, 2002; Arai *et al.*, 2003)

The aCGH technique detects changes in chromosome copy number at a much higher resolution than mCGH (Solinas-Toldo *et al.*, 1997; Pinkel *et al.*, 1998). Instead of using metaphase spreads as the template for hybridization, the aCGH uses a collection of DNA inserts contained in bacterial artificial chromosomes (BAC arrays) or oligonucleotides (oligo-arrays) printed on a glass slide. As with the mCGH, a differentially labeled mixture of DNAs from the test sample and a normal reference control sample is hybridized with the selected platform and the ratio of the fluorescence intensity of the test to reference DNA is calculated. Using aCGH, changes in copy number can be detected based on a few hundred kilobases of DNA sequences for BAC arrays or 30 kb for oligoarrays (Snijders *et al.*, 2001; Fiegler *et al.*, 2003; Medical Genetics Laboratories, 2008). More recently, imbalances involving single nucleotides have been detected by single nucleotide polymorphism (SNP)-based arrays, a type of DNA microarray used to detect polymorphisms within a population (Sherry *et al.*, 2001).

Other variants of FISH technology include multiplex-FISH (M-FISH) and spectral karyotyping (SKY). These procedures allow identification of the origin of each chromosomal region in a metaphase cell by visualizing all 24 human chromosomes in a single hybridization (Speicher and Carter, 2005). In both techniques, the probe set is a pool of differentially labeled DNAs for each of the 24 human chromosomes. In M-FISH, the images for each fluorochrome are collected individually and merged, and a combinatorial labeling algorithm identifies each chromosome that is then visualized in pre-defined pseudocolor (Speicher *et al.*, 1996). In the SKY assay, a single image is captured per cell and an interferometer is used to discriminate the fluorochrome spectrum in each pixel, to which a pseudocolor is assigned (Schröck *et al.*, 1996). Both techniques have been successful in clarifying complex chromosomal rearrangements in solid tumors (Schröck and Padilla-Nash, 2000), including ESCC (Yen *et al.*, 2003).

Together, the numerous variants of FISH technology have allowed the accurate identification of chromosomal DNA sequences of interest and facilitated the screening of the whole genome for gains and losses associated with carcinogenesis.

## Genomic Imbalances In Escc Cell Lines

The few molecular cytogenetic studies of ESCC cell lines reported to date indicate that these cell lines are highly abnormal cytogenetically. In a study in which eight ESCC cell lines were screened using mCGH, SKY and FISH with single probes, the pooled CGH results revealed frequent gains in almost all chromosome arms (1p, 1q, 3q, 5p, 6p, 7p, 7q, 8q, 9q, 11q, 12p, 14q, 15q, 16p, 16q, 17q, 18p, 19q, 20q, 22q and Xq), with frequent losses on 3p, 4, 5q, 6q, 7q, 9p and 18q. SKY analyses detected 195 translocations, 13 deletions and two duplications in the eight cell lines, with the most frequently amplified genes being *PIK3CA* (3q26) and *TP63* (3q28). *PIK3CA* encodes the catalytic subunit of phosphatidylinositol 3-kinase (PIK3), which uses ATP to phosphorylate phosphatidylinositol (National Center for Biotechnology Information, 2009a) and *TP63* encodes a protein involved in the development and maintenance of stratified epithelial tissues (National Center for Biotechnology Information, 2009b). These oncogenes were amplified in six and five cell lines, respectively (Yen *et al.*, 2003).

Multiple gains and losses involving different chromosomal regions were also revealed by mCGH in ten ESCC cell lines of the KYSE series (TE 1-6, 8-11, 13, and 15). The most frequent losses were observed on chromosomal arms 3p, 4p, 4q, 8p, 9p, 18q and Xp, whereas the most common gains were noted on 1q, 3q, 5p, 7p, 8q, 9q, 11q, 18p, 20q and Xq. While focal loss was only identified at 11q23-25, focal amplifications were detect at 1q32, 2p16-22, 3q25-28, 5p13-15.3, 7p12-22, 7q21-22, 8q23-24.2, 9q34, 10q21, 11p11.2, 11q13, 13q32, 14q13-14, 14q21, 14q31-32, 15q22-26, 17p11.2, 18p11.2-11.3 and 20p11.2 (Shinomiya *et al.*, 1999; Pimkhaokham *et al.* 2000; Su *et al.*, 2006).

Yang *et al.* (2008a) recently reported cytogenetic abnormalities in the cell line KYSE 410-4 using M-FISH, with chromosomal gains on 2q, 3, 8, 17p and X. An isochromosome 3q was detected in this line and may represent an intermediate mechanism involved in 3p loss and 3q gain. For the cell line KYSE 180, M-FISH analysis detected loss of DNA copy number on chromosomes 4p, 5q, 6q, 9, 10p, 12p, 13, 14p, 15p, 18p, 18q, 20, 22 and Y, and chromosomal gains and translocations mainly on chromosomes 1, 2p, 3, 4p, 5p, 5q, 6p, 7, 8, 10q, 11, 12q, 14q, 16, 17q, 19 and Xp. Seven derivative chromosomes involving chromosomes 5, 8, 12, 14, 15, 16, and 17 showed complex translocations, each involving three or four chromosomes; a loss of chromosomes 9, 13, and Y was also detected (Wu *et al.*, 2006).

Based on previous CGH studies that showed frequent amplifications in 18p in esophageal cell lines (Shimada *et al.*, 1992; Pimkhaokham *et al.*, 2000), Nakakuki *et al.* (2002) used FISH to screen 29 ESCC cell lines and identified amplifications of 14 known genes and 21 uncharacterized transcripts in chromosome 18 amplicons. These authors also investigated the corresponding levels of gene expression by Southern-, dot- and northern-blotting. Only four known genes (*YES1, TYMS, HEC* and *TGIF*) showed amplification and corresponding over-expression. *YES1* encodes a protein with tyrosine kinase activity, *TYMS* is critical for DNA replication and repair, *HEC* is involved in spindle checkpoint signaling and *TGIF* is a highly conserved transcription regulator with a potential role in the transmission of nuclear signals during development and in adults. These findings suggested that these genes are involved in 18p11.3 amplification and may be associated with esophageal tumorigenesis.

**Figure 1:**
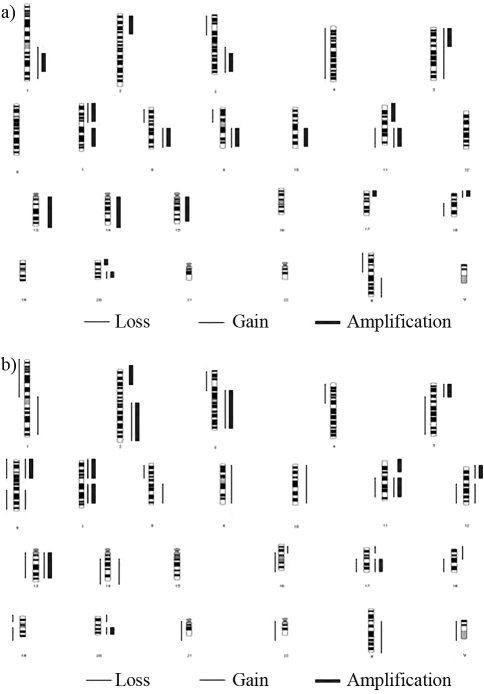
Summary of copy number alterations in esophageal squamous cell carcinomas (ESCC) analyzed by comparative genomic hybridization. Regions with copy number gains are represented by thin lines on the right side of the chromosome idiograms and amplifications are represented by thick bars on the same side; regions of loss are represented by thin lines on the left side of the idiograms. (A) In ESCC cell lines, the most frequent genomic gains were observed in chromosomes 1, 2q, 3q, 5p, 6p, 7, 8q, 9q, 11q, 12p, 14, 15, 16, 17, 18p, 19q, 20q, 22q and X. Focal amplifications were found at 1q32, 2p16-22, 3q25-28, 5p13-15.3, 7p12-22, 7q21-22, 8q23-24.2, 9q34, 10q21, 11p11.2, 11q13, 13q32, 14q13-14, 14q21, 14q31-32, 15q22-26, 17p11.2, 18p11.2-11.3 and 20p11.2. Recurrent losses occurred at 3p, 4, 5q, 6q, 7q, 8p, 9, 10p, 12p, 13, 14p, 15p, 18, 20, 22, Xp and Y. (B) In clinical specimens, chromosomal gains were common in 1q, 2q, 3q, 5p, 6, 7, 8q, 9, 10, 11q, 12, 13q, 14q, 16p, 17, 18q, 20 and Xq, specifically in the regions 1p34, 2p24, 2q24-34, 3q22-ter, 7p12-22, 8q13-qter, 11p11.2, 11q13, 12p11.2, 13q21-34, 17q12, 20q12-13 and Xq27-28. Recurrent losses occurred at 1p, 3q, 4p, 5q, 6, 8p, 11q, 12q, 13q, 14q, 16q, 17q, 18q, 19, 21q, 22q and Y.

Figure 1A summarizes these studies. The alterations detected affected most of the genome and involved regions harboring many known oncogenes and tumor suppressor genes, as well regions not yet associated with such genes. Although the level of molecular resolution of most of these studies is low and inconclusive, these findings are promising in that they provide a starting point for further investigations on the molecular pathogenesis of ESCC and the development of new therapeutic approaches for such cancer.

## Genomic Imbalances In Escc Tumors

Despite advances in our understanding of the risk factors and cellular derangements associated with esophageal cancer, the clinical treatment of this disease remains largely unaltered and long-term survival from this cancer remains poor, with a 5-year survival rate of ~20% (Hsia *et al.*, 2003).

A combination of FISH, mCGH and aCGH has shown that chromosomes 1, 3, 7, 9, 11, 18, 19 and 20 have a high frequency of alterations. In addition, genomic profiles of primary carcinomas have revealed imbalances affecting most of the chromosomes, such as gains on 1q, 3q, 5p, 7p, 8q, 11q, 13q, 18p, 20q and Xq, and losses on 1p, 3p, 4p, 8p, 9p, 18q, 19, 22q and Y. Focal losses at 9p13, focal gains at 5p15, 8p12-11.2, 8q24, 11q13 and 14q32, and amplifications at 1p34, 2p24, 2q24-34, 3q22-ter, 7p12-22, 8q13-qter, 11p11.2, 11q13, 12p11.2, 13q21-34, 17q12, 20q12-13 and Xq27-28 are also commons findings (Pack *et al.*, 1999; Shinomiya *et al.*, 1999; Mayama *et al.*, 2000; Yen *et al.*, 2001; Kamitani *et al.*, 2002; Yen *et al.*, 2003; Kwong *et al.*, 2004; Qin *et al.*, 2004, 2005a,b, 2008; Sugimoto *et al.*, 2007). High-level amplifications have been observed in 30 regions and repeatedly involve 7p11.2 and 11q13. The major gene amplified in the first of these regions is *EGFR*, which encodes the epidermal growth factor receptor (EGFR); activation of this receptor by its ligand results in dimerization and tyrosine auto-phosphorylation that leads to cell proliferation (Carneiro *et al.*, 2008; National Center for Biotechnology Information, 2009c). The second of these regions harbors *CCND1,* a regulator of CDK kinases required for the G1/s cell cycle transition (Carneiro *et al.*, 2008; National Center for Biotechnology Information, 2009d). Interstitial deletions in 1p, 3p, 5q, 6q, 11q and 12q have also been detected (Pack *et al.*, 1999).

Together, these observations indicate that chromosomal aberrations are common in clinical ESCC specimens and suggest that chromosomes 1q, 3q, 5p, 6q, 8q,18p and 20q, particularly regions 1p34, 2p24, 2q24-34, 3q22-ter, 7p12-22, 8q13-qter, 11p11.2, 11q13, 12p11.2, 13q21-34, 17q12, 20q12-13 and Xq27-28, may contain ESCC-related oncogenes; on the other hand, chromosomes 1p, 3p, 4p, 8p, 9p13, 9q and 19p may contain ESCC-related tumor suppressor genes involved in the development and progression of esophageal cancer (Figure 1B).

## Early Genomic Changes and Imbalances Associated With Tumor Staging

ESCC arises through multi-step genetic and cytogenetic alterations. However, the time sequence of these alterations remains to be determined. In this regard, studies in which chromosomal aberrations are correlated with the stage and clinical outcome of prognostic significance are necessary in order to facilitate the selection of patients for specific treatments.

An interesting recent study explored the usefulness of M-FISH for the early diagnosis and risk prediction of precursor lesions of ESCC in tumor and premalignant lesions in 113 patients (Yao *et al.*, 2008). Elevated rates of aneuploidy were frequently observed in chromosomes 3, 8, 10, 12, 17 and 20 in ESCC and its precursor dysplastic lesions. These findings support the conclusion that the application of a multi-target FISH assay to investigate chromosomal aneuploidy at esophageal dysplastic sites may be useful in predicting the risk of ESCC.

The progression of dysplastic lesions to the advanced, metastatic stage is accompanied by numerous genomic changes, as indicated by mCGH analyses of ESCC lymph node metastasis. Copy number gains have frequently been detected at 1q, 1p36.32, 3q, 5p, 8q23-qter, 11q13-14, 5p14-pter, 6p, 20q, 7p22.3, 7q, 2p, 12p, 19p13.3 and 20p, and DNA amplifications have been detected at 11q13, 2q12, 6p12-6q12, 7q21, 20q11.2 and 20p12; losses have been detected at 18q, 3p, 9p, 5q14-23, 4q, 13 and 11q22-qter (Qin *et al.*, 2005a; Wang *et al.*, 2006; Carneiro *et al.*, 2008; Qin *et al.*, 2008).

Gains involving 3q, 5p, 1q and 11q13-14 and losses involving 4 and 13q are significantly correlated with the pathological stage, whereas a gain of 8q and loss of 4p are linked to nodal metastasis; similarly, a gain of 2p and loss of 4 and 11q14-qter are associated with distant organ metastasis (Qin *et al.*, 2004). Gains involving 1q, 3q, 5p and 11q13-14 and losses involving 4 and 13q are critical for the development of ESCC, whereas a gain of 2p and 8q and loss of 4 and 11q14-qter are later events associated with tumor progression and thought to confer metastatic potential to the disease. Nodal and distant organ metastases apparently involve different genes (Qin *et al.*, 2005b). Gains involving 3q and 11q13 and losses involving 3p, 4q, 5q14-23, 9p and 18q have been detected in early and advanced stages of ESCC.

Deletions of 4p and 13q12-q14 and a gain of 5p are significantly correlated with the pathological state. Losses of 8p22-pter and 9p are more frequent in patients with advanced disease. A gain of 8q24-qter is more frequent in patients with grade 3 tumors (Yen *et al.*, 2001). Using mCGH, Shiomi *et al.* (2003) observed that gains involving 3q, 8q, 11q13 and 14q were early events, while the loss of 3p, 5q, 13q and 21q and gain of 1p and Xq were later events in the development of individual tumors.

Gains of 6p and 20p and losses of 10p and 10q are the most significant imbalances in primary carcinoma and metastasis, which suggests that these regions may harbor oncogenes and tumor suppressor genes (Qin *et al.*, 2005a). A gain of 12p and loss of 3p has been associated with poor relapse-free survival (Kwong *et al.*, 2004).

## The Search For Relevant Oncogenes In Escc

In 41 primary ESCC investigated with CGH (Fujita *et al.* 2003), the expression of numerous genes, including the cell cycle-regulator kinase gene *BTAK* (20q13.2-3) and *E2F1* (20q11.2), which plays a crucial role in cell cycle regulation, was enhanced in ~10% of tumors. Other genes, such as *NCOA3* (20q12), which encodes a nuclear receptor co-activator that enhances the transcriptional activator function of nuclear hormone receptors, and *DcR3* (20q13.3), which regulates apoptosis were up-regulated to a lesser extent. Xu *et al.* (2007) detected *NCOA3* overexpression and an increased copy number in 46% and 13% of 221 ESCCs, respectively. *NCOA3* overexpression was observed more frequently in late compared to early stages, but there was no significant association between the expression of *NCOA3* and lymph node metastases. These observations suggest that overexpression of *NCOA3* as a result of genomic gain or other molecular mechanisms might provide a selective advantage for the development and local invasion of certain subsets of ESCC.

To date, there has been no detailed analysis of loss of heterozygosity (LOH) for chromosome 18q in ESCC. However, LOH on chromosome 18q is common in several cancers, with frequencies of 55%-67% in colorectal cancer (Jen *et al.*, 1994; Thiagalingam *et al.*, 1996), 90% in pancreatic cancer (Hahn *et al.*, 1996), 59% in ovarian cancer (Lassus *et al.*, 2001) and 40%-84% in head and neck squamous cell carcinoma (Papadimitrakopoulou *et al.*, 1998; Pearlstein *et al.*, 1998; Takebayashi *et al.*, 2000). In these cancers, frequent LOH on chromosome 18q correlates with tumor growth, aggressive tumor behavior and tumorigenesis. These findings suggest that chromosome 18q may harbor tumor suppressor genes for various cancers (Ando *et al.*, 2007). In a FISH analysis of the allelic imbalance of chromosome 18q in ESCC resected samples from two out of five patients showed a loss of one copy of chromosome 18q, and 13 of 46 ESCC samples (28.3%) showed loss of almost all of chromosome 18q (Ando *et al.*, 2007). The authors suggested that the loss of 18q may play an important role in the progression of ESCC.

Few studies have focused on the identification of altered pathways and clinically applicable markers. Oncogene amplification was examined by DNA microarrays in 20 surgically resected ESCC and 57 oncogenes were found to be amplified. Alterations in DNA copy number detected by microarrays were compared to those obtained by mCGH. DNA microarrays showed that eight oncogenes (*CCND1*, *FGF3/ FGF4*, *EMS1*, *SAS*, *ERBB2*, *PDGFRA*, *MYC* and *BCL2*) were amplified in 9 of 20 tumors. Although *ERBB2* expression was 23-fold greater than the basal level in one case, the average level of gene amplification was generally only 2-4-fold above the control value. *EMS1,CCND1* and *FGF3/FGF4,* which are all located on 11q13, were amplified in 7, 5 and 4 of 20 ESCC, respectively, and were co-amplified in three tumors. *EMS1* regulates the interactions between components of adherens-type junctions and organizes the cytoskeleton and cell adhesion structures of epithelia and carcinoma cells (National Center for Biotechnology Information, 2009e). *FGF3/FGF4* broadly regulate mitogenesis, cell survival and oncogenic activity.

A comparison of genomic DNA microarray and mCGH data showed that although most of the amplified oncogenes were included in chromosomal regions for which gains in DNA copy number were detected by mCGH, not all of the amplified genes detected by microarrays showed concomitant gains in the DNA copy number in mCGH; this lack of correlation between the two techniques confirmed the limited resolution of the mCGH assay. Microarrays of oncogenes are useful for the comprehensive identification of amplified oncogenes and for analyzing specific chromosomal regions in which mCGH analysis indicates an increase in DNA copy number (Arai *et al.*, 2003).

FISH assays revealed the amplification of *PLK1* (polo-like kinase 1), an essential gene for the maintenance of genomic stability during mitosis (Feng *et al.*, 2009); this gene may therefore be a useful prognostic marker. An analysis of 108 ESCCs and nine ESCC cell lines revealed a frequent gain of the genes *MDS1* (myelodysplasia syndrome 1) and *PRKCI* (protein kinase C, iota), which have been implicated in neoplastic transformation, and a positive correlation between the level of *PRKCI* expression and tumor size, lymph node metastasis and clinical stage. *PRKCI* gene amplification was highly correlated with protein overexpression (Yang *et al.*, 2008b).

EGFR expression is enhanced in many cancers and is sometimes accompanied by gene amplification. FISH analysis has shown that the *EGFR* gene is amplified in ESCC (Sunpaweravong *et al.*, 2005; Hanawa *et al.*, 2006). Indeed, *EGFR* gene overrepresentation (balanced gene and chromosome 7 polysomy) and *HER-2* amplification are common events in ESCC (Mimura *et al.*, 2005; Sunpaweravong *et al.*, 2005; Bizari *et al.*, 2006).

Cyclin D1 (*CCDN1*) is a cell-cycle regulator and oncogene implicated in the pathogenesis of numerous types of tumors. Amplification of the *CCDN1* gene is common in ESCC and has been detected by FISH (Sheyn *et al.*, 1997; Jin *et al.*, 2004; Manoel-Caetano *et al.*, 2004; Sunpaweravong *et al.*, 2005; Bizari *et al.*, 2006).

The correlation between the overexpression and amplification of oncogenes and patient survival has not been extensively investigated. However, the survival of patients with increased expression of *BTAK* or *E2F1* is significantly lower than that of patients without this alteration, which suggests that overexpression of these genes probably increases the number of malignant ESCC phenotypes and may be a useful marker of poor prognosis (Fujita *et al.*, 2003).

## Concluding Remarks

Various molecular cytogenetic approaches have been used to demonstrate the extensive genetic complexity associated with the different stages of ESCC and have identified key genes involved in esophageal cancer. Most of the unbalanced chromosomal regions are similar in primary tumors and cell lines, thus confirming that cell lines provide a reliable model for investigating the molecular mechanisms involved in ESCC.

Specific chromosomal imbalances are associated with the progression of esophageal tumors. Whereas gains in 1q, 5p, 8q, 14q and losses in 4p, 13q and 18q are associated with the early stages of ESCC development, gains in 1p, 2p, 7p22.3, 8q, 8q24-qter and Xq and losses in 8p22-pter, 11q14-qter, 13q and 21q are associated with advanced stages. Gains involving 3q and 11q13 and losses involving 3p, 4q, 5q14-23, 9p and 18q are detected in both early and advanced stages of ESCC. These regions harbor genes associated with essential cellular processes, such as signal transduction, transcriptional regulation, cell proliferation and cell differentiation, involved in signalizing cascades related to cancer. Some imbalances, such as losses involving 4p, 9p and 11q and gains involving 1p36-32, 5p, 7q and 19p13.3, are poor prognostic markers. Gene overexpression most frequently affects oncogenes such as *YES1,TYMS, HEC, TGIF, NCOA3, BTAK, DCR3, E2F1, MYC, EGFR, EGR2, CCND, FGF3/FGF4, EMS1, SAS, ERBB2, PDGFR1, BCL2, MDS1* and *PRKCI*, whereas genomic losses lead to the deletion of suppressor tumor genes such as *CDKN2A, MTAP* and *TP53*. Identification of the role of these critical genes in esophageal carcinogenesis will assist the development of individualized target therapy that should in turn improve the clinical outcome of ESCC patients.
